# A paradoxical relationship between Resveratrol and copper (II) with respect to degradation of DNA and RNA

**DOI:** 10.12688/f1000research.7202.2

**Published:** 2016-03-02

**Authors:** Siddharth Subramaniam, Iqbal Vohra, Aishwarya Iyer, Naveen K Nair, Indraneel Mittra

**Affiliations:** 1 Translational Research Laboratory, Advanced Centre for Treatment, Research and Education in Cancer, Tata Memorial Centre, Kharghar, Navi-Mumbai, 410210, India

**Keywords:** Resveratrol, copper, pro-oxidant activity, plasmid DNA cleavage, plasmid DNA degradation, eukaryotic DNA degradation, RNA degradation

## Abstract

Resveratrol (R), a plant polyphenol, is known to reduce Cu (II) to Cu (I) generating reactive oxygen species that can cleave plasmid DNA. Here we report a surprising observation of a paradoxical relationship between R and Cu whereby plasmid DNA cleaving / degrading activity of R-Cu increased progressively as the ratio of R to Cu was increased i.e., the concentration of Cu was successively reduced with respect to a fixed concentration R. Whereas cleavage of plasmid DNA occurred at low molar ratios of R to Cu, at higher ratios, complete degradation of DNA was achieved. By further increasing the ratio, whereby the concentration of Cu was reduced to very low levels, the DNA degrading activity of R-Cu was lost. This paradoxical relationship is also seen with respect to eukaryotic genomic DNA and RNA. Since R-Cu may have anti-cancer and anti-viral activities, our findings may not only help to improve the therapeutic efficacy of R-Cu but also reduce its toxic side effects with the use of low concentration of Cu.

## Introduction

Resveratrol (R) is a poly-phenolic stilbenoid naturally present in the skin of red grapes and other fruits and berries, peanuts and also in the roots of Japanese knotweed ^1^. R has been shown to have multiple health benefits that include life extension, cancer prevention, cardio-protection, neuro-protection and anti-diabetic, anti-inflammatory and anti-viral activities ^2– 8^. These actions are thought to be mediated through its intrinsic anti-oxidant properties and the ability of R to activate SIRT1 ^9– 11^. However most of the positive effects exhibited by R could not be replicated in clinical trials possibly because of its low bio-availability ^12, 13^.

Copper (Cu) is an essential micronutrient, and because of its role as a metal co-factor, has the ability to generate reactive oxygen species (ROS), *viz*., O _2_^-.^ and ^**•**^HO radicals ^14^. Fukuhara and Miyata were first to show that R can act as a pro-oxidant in the presence of Cu and cause oxidative DNA cleavage in a pBR322 plasmid assay ^15^. R forms a complex with Cu (II), leading to its reduction to Cu (I) with concomitant production of ROS which is responsible for DNA scission ^16^. Resveratrol-copper (R-Cu) was shown to be active in biological systems as evidenced by its ability to inactivate bacteriophages ^8^ and to cause fragmentation of DNA of human lymphocytes *in vitro* ^17^. These findings have led to the proposal that R-Cu could be used in the prevention and treatment of cancer ^17, 18^.

The above studies have used variable molar ratios of R:Cu which have usually been of the order of 1:1 to 2:1. Here we report a surprising observation that DNA and RNA cleaving and/or degrading activity of R-Cu increases as the ratio of R to Cu is sequentially increased (i.e., the concentration of Cu is sequentially decreased with respect to a fixed concentration of R). The activity was lost when the Cu concentration was reduced to very low levels.

## Methods

### Isolation of DNA and RNA

***Isolation of plasmid pTRIPZ DNA.*** Isolation of plasmid *pTRIPZ* DNA was performed using HiPurA plasmid DNA miniprep purification spin kit (Hi-Media) as per manufacturer’s instructions. Briefly, the transformed bacterial culture ( *Escherichia coli* DH _5_α containing plasmid *pTRIPZ*. Invitrogen, USA) was harvested, lysed and centrifuged. The pellet obtained was applied to a silica column and high salt (3M Potassium acetate, pH 5.5) was used to allow binding of plasmid DNA to the silica column. Washing for removal of contaminants was followed by elution of plasmid DNA in DNA binding buffer.

***Eukaryotic genomic DNA.*** Jurkat (human lymphoblastic leukemia) cells were used for isolation of genomic DNA. Cells were procured from American Type Culture Collection and were grown in RPMI 1640 (GIBCO By Life technologies Cat No.23400-21) with 10% FBS (GIBCO By life technologies Cat No.26140-079). The Wizard® Genomic DNA purification kit (Promega) was employed for isolation of DNA. Jurkat cells (2 × 10 ^6^) were harvested and given three PBS washes followed by treatment with nuclei lysis solution. Genomic DNA was isolated as per manufacturer’s protocol.

***Isolation of eukaryotic RNA.*** Jurkat cells at the exponential phase of growth (approximately 5 × 10 ^6^) were washed thrice in PBS and RNA was isolated using Trizol® reagent (Life Technologies, Carlsbad, CA, USA) as per manufacturer’s protocol.

### Preparation of Resveratrol-Cu reaction mixture and gel electrophoresis

Stock solutions of Resveratrol (Sigma-Aldrich) (20mM) and that of CuSO _4_.5H2O (MP Biomedical) (20mM) were prepared in 60% ethanol and water respectively. The reaction mixture contained a fixed amount of R and varying amounts of Cu (as specified in the text) and 500ng of plasmid or genomic DNA or 2µg RNA in a sterilized 1.5 ml micro-tube. Volume of the mixture was kept constant at 20 µl (5µl of R, 5µl of Cu and 10µl of DNA). Reaction mixtures were prepared containing varying starting concentrations of R as follows: 100µM, 500µM, 1mM and 5mM (see text). The mixture was incubated at 37°C for 1 hr. In case of plasmid and genomic DNA, electrophoreses was performed on a 1% agarose gel using a horizontal electrophoresis unit (Hoefer) at a constant voltage of 100V. In case of eukaryotic RNA, the mixture was electrophoresed on a 0.8% agarose gel at 75 volts for 90 minutes. The gel-documentation system - EC-3 Imaging system from UVP (Ultra Violet Products, USA) was used to record the images.

## Observation

**Figure 1.**
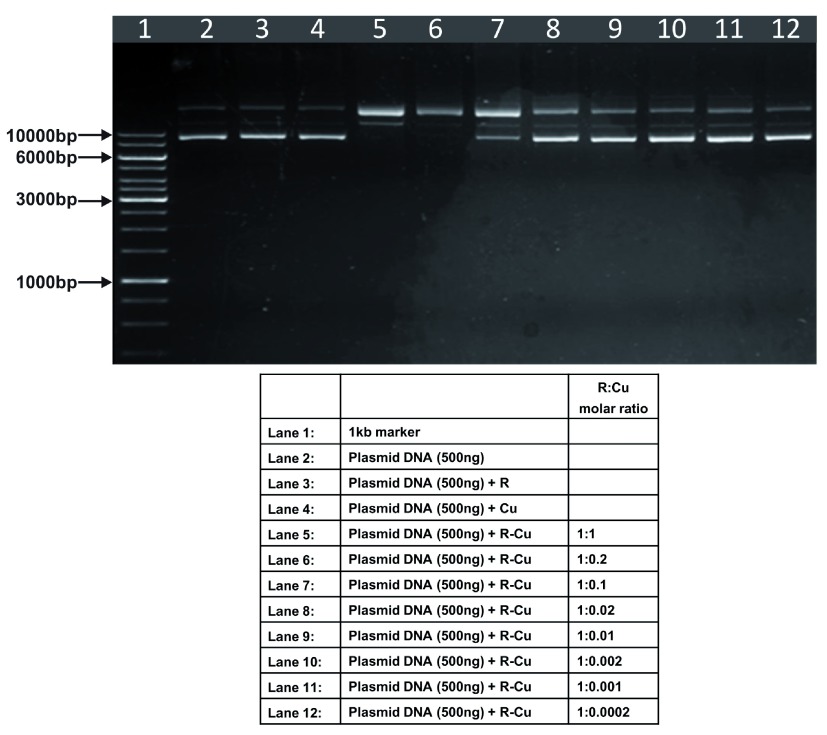
Increasing cleavage/degradation of plasmid DNA by R-Cu in the presence of decreasing concentrations of Cu. Starting concentration R 100µM:Cu 100µM. Reactions were performed in 50% ethanol.

**Figure 2.**
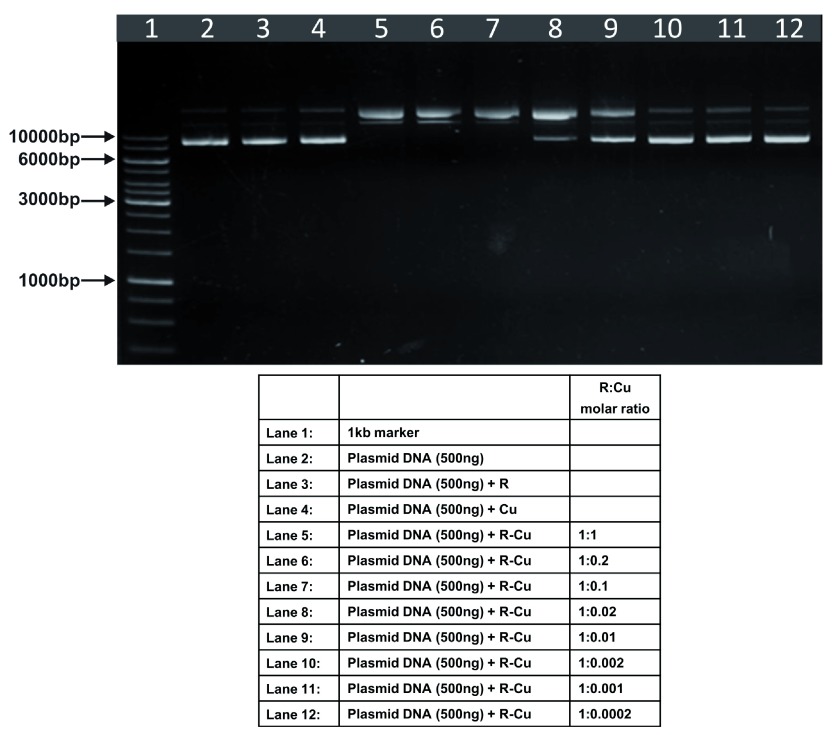
Increasing cleavage/degradation of plasmid DNA by R-Cu in the presence of decreasing concentrations of Cu. Starting concentration R 500µM:Cu 500µM. Reactions were performed in 50% ethanol.

**Figure 3.**
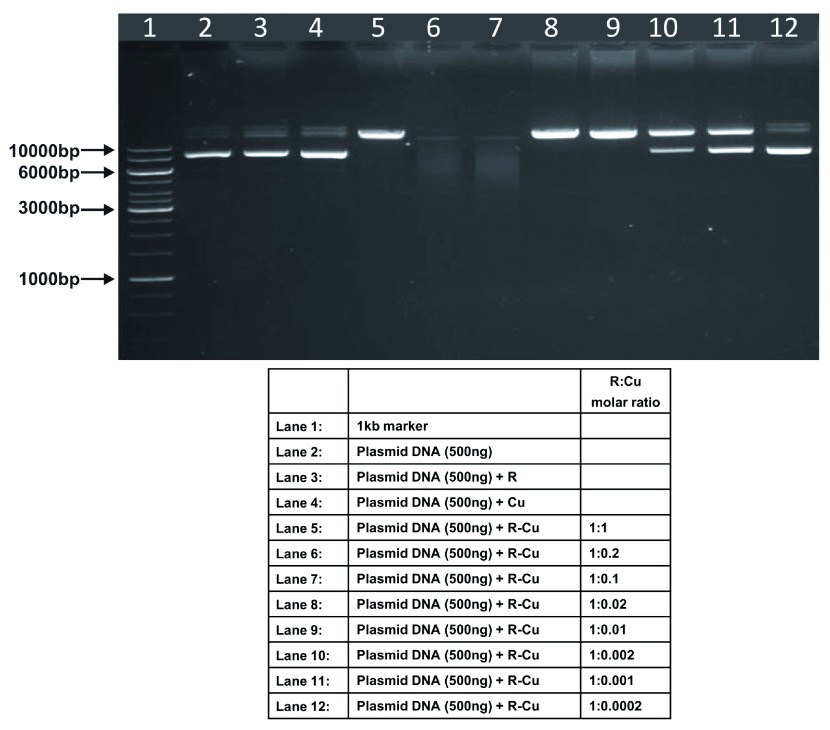
Increasing cleavage/degradation of plasmid DNA by R-Cu in the presence of decreasing concentrations of Cu. Starting concentration R 1mM:Cu 1mM. Reactions were performed in 50% ethanol.

**Figure 4.**
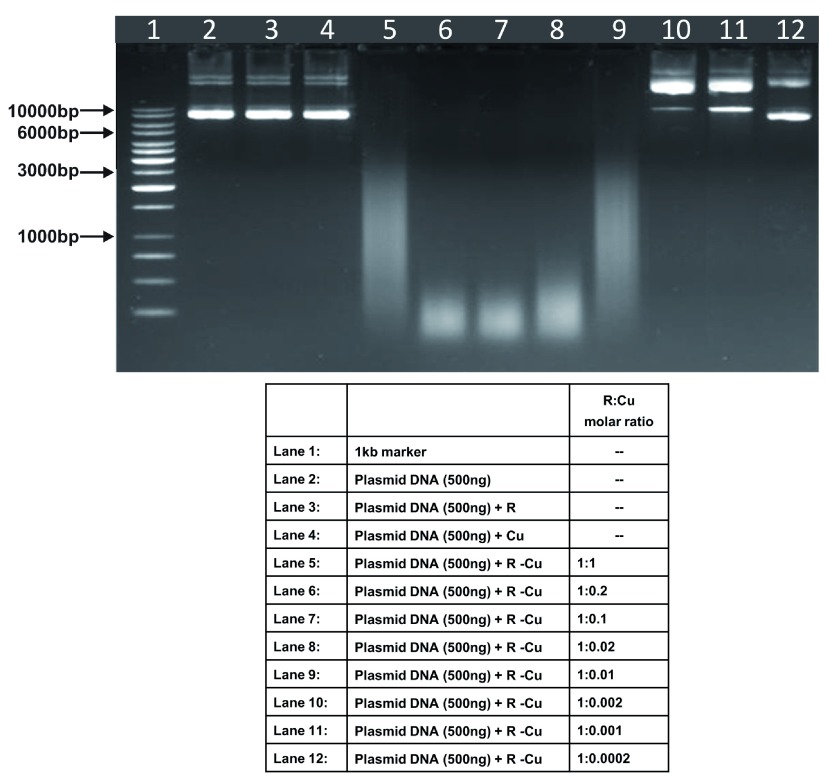
Increasing cleavage/degradation of plasmid DNA by R-Cu in the presence of decreasing concentrations of Cu. Starting concentration R 5mM:Cu 5mM. Reactions were performed in 50% ethanol.

**Figure 5.**
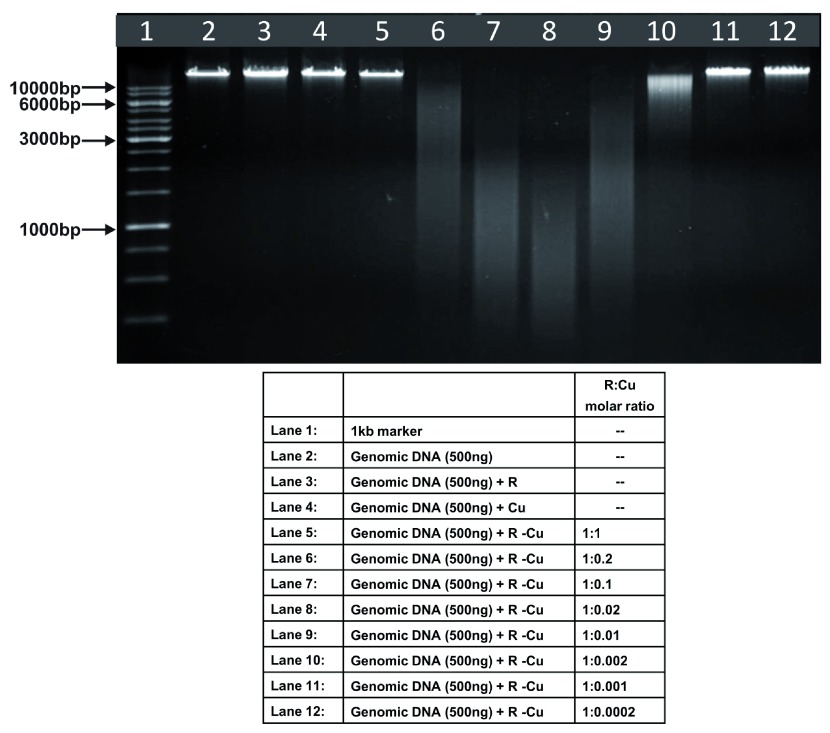
Increasing cleavage/degradation of eukaryotic genomic DNA by R-Cu in the presence of decreasing concentrations of Cu. Starting concentration R 5mM:Cu 5mM. Reactions were performed in 50% ethanol.

**Figure 6.**
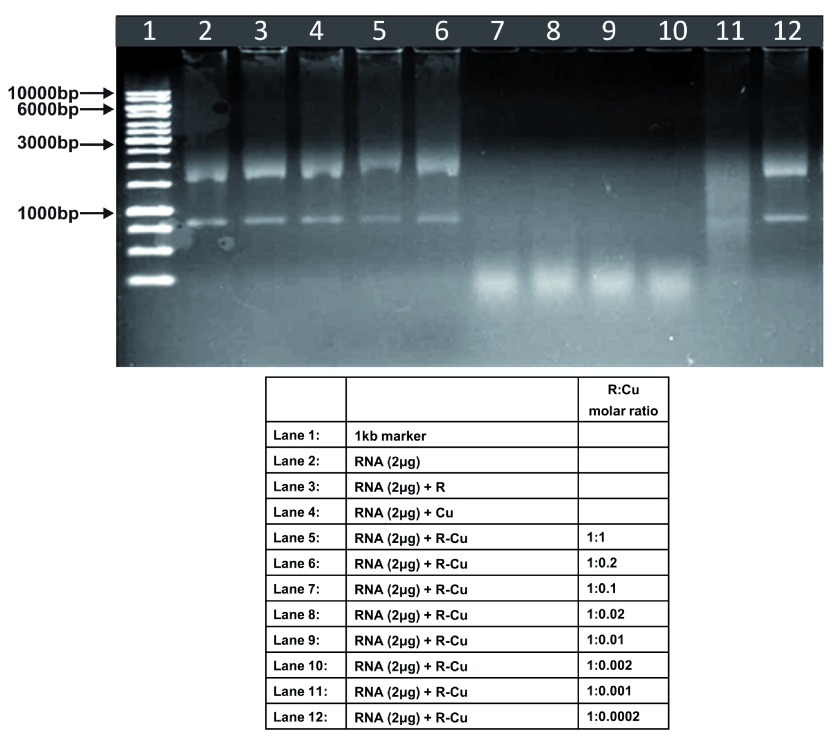
Increasing cleavage/degradation of eukaryotic RNA by R-Cu in the presence of decreasing concentrations of Cu. Starting concentration R 5mM:Cu 5mM. Reactions were performed in 50% ethanol.

**Figure 7.**
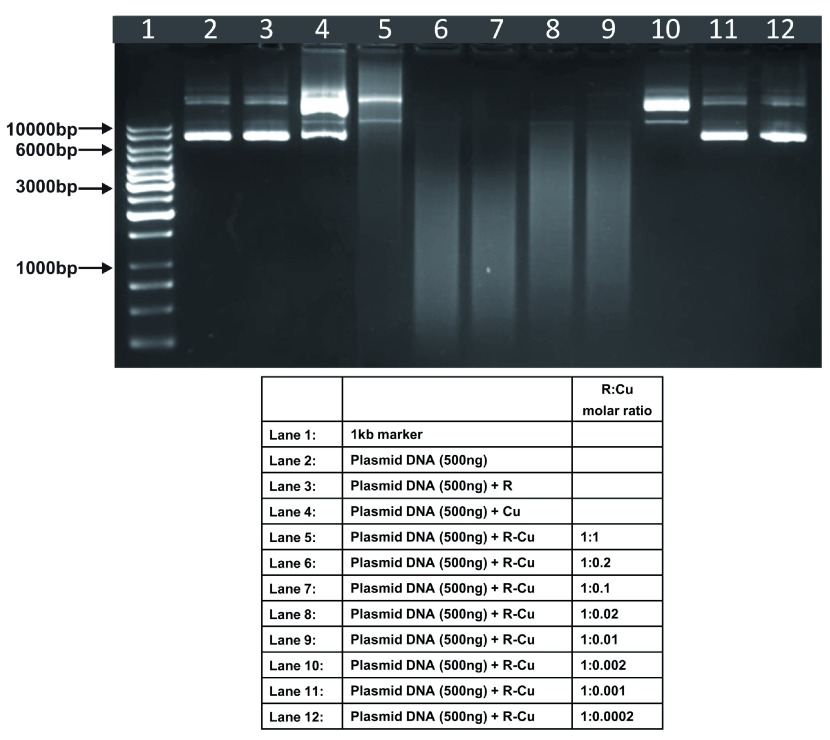
Increasing cleavage/degradation of plasmid DNA by R-Cu in the presence of decreasing concentrations of Cu in different solvents. Reactions were performed in 50% Acetonitrile.

The table summarizes our observations under 3 separate headings: 1) observations on plasmid DNA using reducing concentrations of Cu and a constant concentration of R ( Figure 1– Figure 4); 2) observations on eukaryotic DNA and RNA with reducing concentrations of Cu and a constant concentration of R ( Figure 5, Figure 6), and 3) observations on plasmid DNA using different solvents with reducing concentrations of Cu and a constant concentration of R ( Figure 7– Figure 9).

**Figure 8.**
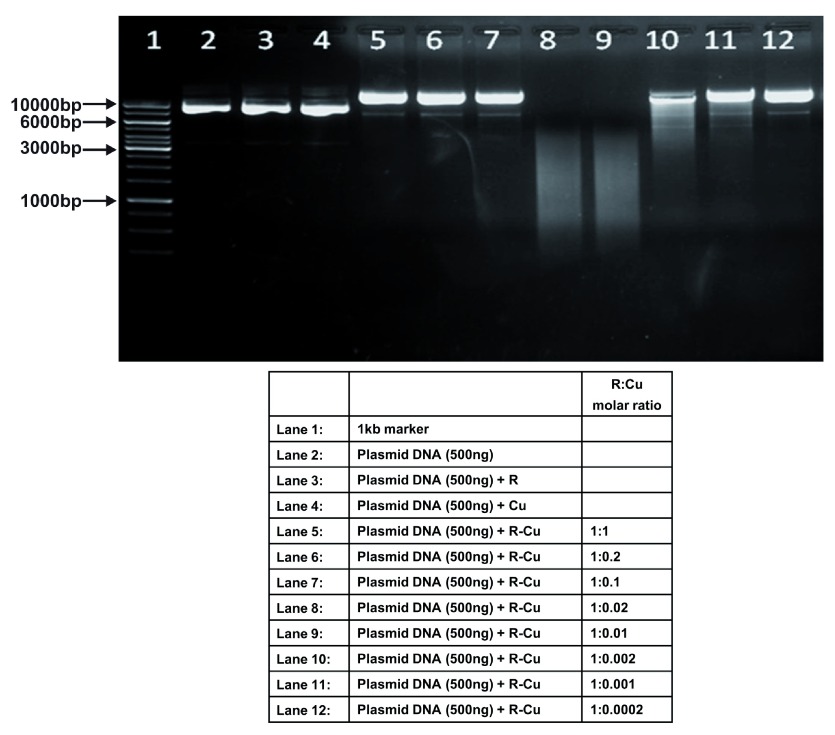
Increasing cleavage/degradation of plasmid DNA by R-Cu in the presence of decreasing concentrations of Cu in different solvents. Reactions were performed in 3mM NaOH.

**Figure 9.**
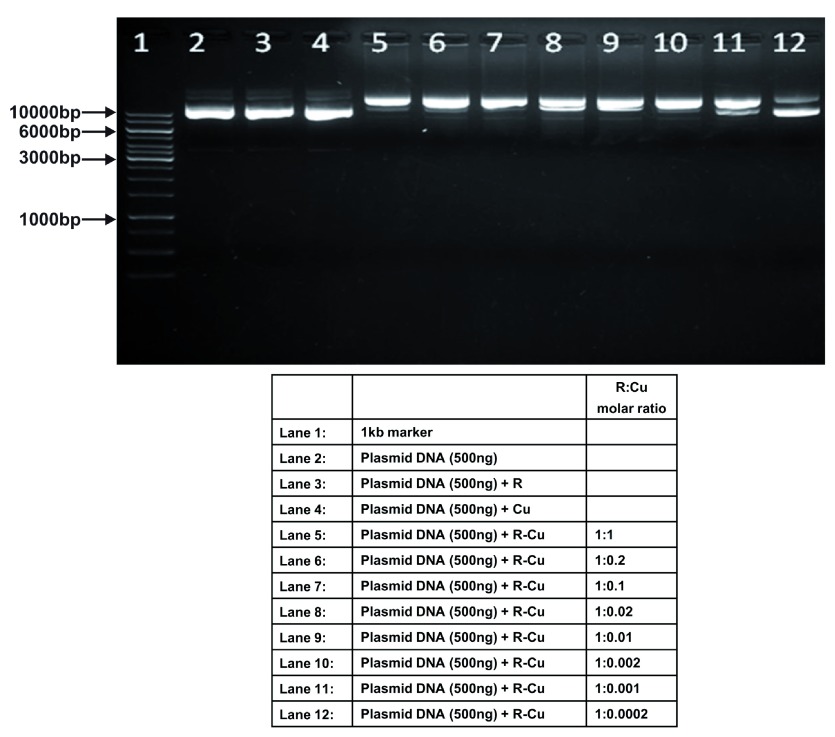
Increasing cleavage/degradation of plasmid DNA by R-Cu in the presence of decreasing concentrations of Cu in different solvents. Reactions were performed in water.

We observed that when we increased the ratio of R to Cu (by reducing the concentration of Cu with respect to a fixed concentration of R) there was an enhancement of cleavage/degradation of plasmid DNA ( Figure 1– Figure 4). This phenomenon was dependent on the starting concentration of R-Cu. For example, cleavage of supercoiled plasmid DNA was observed at a starting concentration of 100μM at molar ratios of 1:1 and 1:0.2 (lanes 5 and 6; Figure 1 and Table). However, with successive increases in starting concentration of R-Cu to 500μM, 1mM and 5mM, DNA cleaving activity was progressively enhanced such that complete cleavage was achieved at successively higher ratios of R to Cu (i.e., with decreasing Cu concentration) ( Figure 2– Figure 4 and Table). At high starting concentrations *viz*., 1mM and 5mM, degradation rather than cleavage of DNA was observed. These data indicated that the DNA cleaving/degrading activity of R-Cu increases as the ratio of R to Cu is successively increased thereby suggesting the existence of a paradoxical relationship between R and Cu with respect to DNA cleavage/degradation. The data also show that the extent of cleavage/degradation is positively correlated with the starting concentrations of R and Cu. Figure 5 and Figure 6, in which genomic DNA and RNA respectively were used (starting molar ratio of R to Cu of 5mM:5mM), a similar paradoxical pattern was observed ( Table).

**Table.** Plasmid DNA (in 50% ethanol): Variable starting concentrations ( Figure 1– Figure 4).

Sr No	Starting Concentration	Cleavage/Degradation									
		R	Cu	1:1	1:0.2	1:0.1	1:0.02	1:0.01	1:0.002	1:0.001	1:0.0002
1	100µM: 100µM	-	-	✔	✔	✔	-	-	-	-	-
2	500µM: 500µM	-	-	✔	✔	✔	✔	-	-	-	-
3	1mM: 1mM	-	-	✔	✔✔✔	✔✔✔	✔	✔	-	-	-
4	5mM: 5mM	-	-	✔✔✔	✔✔✔	✔✔✔	✔✔✔	✔✔✔	✔	✔	-

**Table:** Genomic DNA and RNA (in 50% ethanol): Starting concentration 5mM:5mM ( Figure 5, Figure 6).

Sr No	Substrate	Cleavage/Degradation									
		R	Cu	1:1	1:0.2	1:0.1	1:0.02	1:0.01	1:0.002	1:0.001	1:0.0002
1	Genomic DNA	-	-	-	✔✔✔	✔✔✔	✔✔✔	✔✔✔	✔✔✔	✔	-
2	RNA	-	-	-	-	✔✔✔	✔✔✔	✔✔✔	✔✔✔	✔✔✔	-

**Table:** Plasmid DNA (in various solvents): Starting concentration 5mM:5mM ( Figure 4; Figure 7– Figure 9).

Sr No	Solvent	Cleavage/Degradation									
		R	Cu	1:1	1:0.2	1:0.1	1:0.02	1:0.01	1:0.002	1:0.001	1:0.0002
1	50% Ethanol	-	-	✔✔✔	✔✔✔	✔✔✔	✔✔✔	✔✔✔	✔	✔	-
2	50% Acetonitrile	-	-	✔	✔✔✔	✔✔✔	✔✔✔	✔✔✔	✔	-	-
3	3mM NaOH	-	-	✔	✔	✔	✔✔✔	✔✔✔	✔	✔	✔
4	Water	-	-	✔	✔	✔	✔	✔	✔	✔	-

The above experiments were done in 50% ethanol ( Figure 1– Figure 4). We undertook similar experiments under different solvent conditions, namely, 50% acetonitrile ( Figure 7), 3mM NaOH ( Figure 8) and water ( Figure 9). We observed a similar paradoxical relationship under all three conditions ( Table). Cleavage of plasmid DNA was most efficient in 50% acetonitrile wherein cleavage was seen to commence at a R:Cu ratio of 1:1 while complete degradation occurred in all ratios between 1:0.2 and 1:0.01. Cleavage/degradation was less efficient in 3mM NaOH wherein cleavage of plasmid DNA was seen between 1:1 and 1:0.1; complete degradation being observed at ratios of 1:0.02 and 1:0.01. Water proved to be the least efficient medium where degradation was not seen under any R-Cu ratios although cleavage was observed at all ratios of R-Cu between 1:1 and 1:0.0002. The above findings suggested that reduction of Cu(II) to Cu(I) to generate free radicals can occur under diverse conditions leading to cleavage/degradation of DNA.

## Discussion

Spectroscopic studies using an analogue of Resveratrol, namely Piceatannol (3,3',4,5'-tetrahydroxy-trans-stilbene; Pice), have shown that Pice-Cu(II) induced DNA cleavage involves the Haber Weiss and Fenton reactions ^19^. DNA cleavage is a result of hydroxyl radical formation and the Cu (II) to Cu (I) redox cycle generated ROS production ^19^. Our experiments using R-Cu reported here suggest that the Cu (II) – mediated oxidation of R is in a catalytic mode via Cu (II) – Cu (I) redox cycling; Cu (II) acts as a catalyst with an optimum dosage depending on the starting concentration of R-Cu. However, our unexpected finding of increasing DNA and RNA cleavage/degradation with decreasing concentration of Cu remains currently unexplained and requires further investigation.

Since R-Cu may have anti-cancer and anti-viral activities ^8, 18^, our finding may not only help to improve the therapeutic efficacy of R-Cu but also reduce its toxic side effects with the use of low concentration of Cu.
