# Supplementary material for: A paradoxical relationship between Resveratrol and copper (II) with respect to degradation of DNA and RNA
Source: F1000Res. 2016 Mar 2;4:1145. Originally published 2015 Oct 27. [Version 2] doi: 10.12688/f1000research.7202.2 (PMC4833056; doi:10.12688/f1000research.7202.2)
Supplement: Peer review history

# Peer review: Referee response for version 2

**DOI:** 10.5256/f1000research.8442.r13360

**Published:** 2016-04-14

**Authors:** Asfar S. Azmi

**Affiliations:** 1 Department of Oncology, School of Medicine, Karmanos Cancer Institute, Wayne State University, Detroit, MI, USA

The studies in this article demonstrate that the requirement of Cu becomes minimal with increasing concentrations of resveratrol in mediating DNA damage to plasmid DNA. These results are in line with previous reports where copper-resveratrol complex was shown to induce oxidative DNA breakage in plasmid DNA as well as in human peripheral lymphocytes. The article is well written, however, it can be enhanced by the addition of a paragraph on the bioavailability of resveratrol. This is especially important given that the doses being used to show DNA cleavage are quite high to be pharmacologically achievable. Will such high concentrations be achieved *in vivo*? Also is there a saturating concentration where the role of Cu becomes insignificant and resveratrol alone induces oxidative DNA damage. Addition of these points will certainly enhance the readability of the manuscript.

I have read this submission. I believe that I have an appropriate level of expertise to confirm that it is of an acceptable scientific standard.

# Peer review: Referee response for version 2

**DOI:** 10.5256/f1000research.8442.r12852

**Published:** 2016-03-10

**Authors:** Renata Cozzi

**Affiliations:** 1 Department of Science, Roma Tre University, Rome, Italy

I have read the new version of the manuscript and I find that the authors have properly answered to my comments and requests.

I have read this submission. I believe that I have an appropriate level of expertise to confirm that it is of an acceptable scientific standard.

# Peer review: Referee response for version 1

**DOI:** 10.5256/f1000research.7758.r11205

**Published:** 2015-11-12

**Authors:** Renata Cozzi

**Affiliations:** 1 Department of Science, Roma Tre University, Rome, Italy

The topic of this paper is very important and has interesting implications for the purpose of using resveratrol  as chemopreventive agent. In fact the authors rightly refer to the paper by Azmi *et al. *where the problem of the potential breaking activity of resveratrol in human lymphocytes is addressed.

However in my opinion the interpretation of the results of this manuscript is not always supported by the data presented.

Referring to figures from 1 to 4 the authors state: “with successive increases in starting concentration of R-Cu to 500μM, 1mM and 5mM, DNA cleaving  activity was progressively enhanced such that complete cleavage was achieved at successively higher ratios of R to Cu”. This is partially true in the sense that starting from the ratio R-Cu 1:1 the cleavage of supercoiled increases until 1:0.01 with the highest dose of resveratrol (5mM).  At lower ratios no cleavage/degradation  is observed. In other word it seems that the cleaving activity is due to resveratrol concentration increase rather than the ratio R-Cu.

In fact the authors confirm this reading of the results (“The data also show that the extent of cleavage/degradation is positively correlated with the starting concentrations of R and Cu”) but suggest also  “the existence of a paradoxical synergistic relationship” (even in the title) that is not at all supported by the data. The word “synergism” does not seem appropriate.

As far as eukaryotic genomic DNA a similar behavior is showed: the cleavage/degradation of DNA is evident from ratio 1:0.2 to 1:0.002,not at lower ratios.

I suggest:

1. To use (certainly in the title and throughout  the text) a less demanding word than “synergism”. Furthermore a synergistic effect would require also a statistical evaluation.
2. To provide an explanation and/or an interpretative hypothesis about the showed effect present only in the middle ratios.
3. To provide a more accurate description of figure 7, 8 and specially 9.

I have read this submission. I believe that I have an appropriate level of expertise to confirm that it is of an acceptable scientific standard, however I have significant reservations, as outlined above.

# Response

**Published:** 2016-01-14

**Authors:** Indraneel Mittra

**Affiliations:** Tata Memorial Centre, India

1. As suggested by the Reviewer, we have replaced the word "synergism" with "relationship" in the title and throughout the paper.
2. As mentioned in the 'discussion' section, we are unable to provide any explanation for this paradoxical relationship between Resveratrol and copper. This is the reason why we have submitted the manuscript under "Observation" section of the Journal which stipulates "We welcome Observation Articles describing a novel observation that may be unexpected, and possibly currently without explanation". We would be delighted if the Referee could provide us some clues.
3. We have now provided an accurate descriptions of Figures 7, 8 and 9.
  In order for the reader to easily grasp our findings depicted in multiple gel pictures, we have provided a 'summary table' of all the gel figures in a tabular format (Table) which reflects the paradoxical relationship between R and Cu more clearly. 

We trust that we have satisfactorily responded to all the suggestions of the referee and hope that she would now find the paper worthy of approval.
